# Continuous IL-23 stimulation drives ILC3 depletion in the upper GI tract and, in combination with TNFα, induces robust activation and a phenotypic switch of ILC3

**DOI:** 10.1371/journal.pone.0182841

**Published:** 2017-08-08

**Authors:** Amanda M. Schmidt Paustian, Jesus Paez-Cortez, Shaughn Bryant, Susan Westmoreland, Wendy Waegell, Gillian Kingsbury

**Affiliations:** AbbVie Bioresearch Center, Worcester, MA, United States of America; McGill University, CANADA

## Abstract

Mutations in the Interleukin (IL)-23/IL-23 receptor loci are associated with increased inflammatory bowel disease (IBD) susceptibility, and IL-23 neutralization has shown efficacy in early clinical trials. To better understand how an excess of IL-23 affects the gastrointestinal tract, we investigated chronic systemic IL-23 exposure in healthy wildtype mice. As expected, IL-23 exposure resulted in early activation of intestinal type 3 innate lymphoid cells (ILC3), followed by infiltration of activated RORγt+ T helper cells. Surprisingly, however, sustained IL-23 stimulus also dramatically reduced classical ILC3 populations within the proximal small intestine, and a phenotypically distinct T-bet expressing ILC3 population emerged. TNFα neutralization, a widely used IBD therapy, reduced several aspects of the IL-23 driven ILC3 response, suggesting a synergy between IL-23 and TNFα in ILC3 activation. *In vitro* studies supported these findings, revealing previously unappreciated effects of IL-23 and TNFα within the intestine.

## Introduction

Interleukin (IL)-23 has been associated with the development of several debilitating autoimmune diseases, including psoriasis and inflammatory bowel disease (IBD) [1]. Therapeutic IL-23 neutralization has recently proven efficacious for both indications, but the pathogenic role of IL-23 within the gastrointestinal (GI) tract remains poorly understood [2].

Studies performed in IL-23 receptor (IL-23R) reporter mice have shown that T cells and innate lymphoid cell (ILC) populations residing within the lamina propria (LP) are the primary responders to IL-23 in the healthy GI tract [3]. Several murine studies have found IL-23 responsive RORγt+ type 3 innate lymphoid cells (ILC3) are a critical component of the intestinal immune response [4, 5], but how these cells respond to chronic IL-23 stimulation has not been carefully studied.

Here we utilized a previously described method of DNA minicircle (mc) injection to drive sustained systemic expression of murine IL-23 in healthy adult mice [6, 7]. Examination of intestinal pathology in IL-23 mc injected mice revealed inflammation and lengthening of the small intestine (SI); and a dramatic activation and loss of RORγt+ ILC3 populations from the LP was observed. While both CCR6+ and Nkp46 expressing (NCR+) ILC3 populations were rapidly depleted, cytokine production by remaining ILC3 was enhanced and a CCR6-, NCR-, T-bet+, IL-7R-, ILC3 population emerged. Prophylactic treatment with anti-TNFα antibody did not alter this ILC3 loss, but significantly inhibited ILC3 cytokine production and prevented the outgrowth of the IL-23 mc elicited ILC3 subset. Additionally, *in vitro* stimulation of sorted intestinal ILCs found TNFα to synergize with IL-23 to drive robust activation of ILC3. To our knowledge, this is the first data showing that (1) chronic IL-23 stimulation leads to a depletion of classical ILC3 populations from the proximal SI, and (2) TNFα directly promotes IL-23-driven ILC3 activation.

## Materials and methods

### Mice

8–16 week old female C57BL/6J (B6) mice (Jackson Laboratories) and B6.129S6-Rag2tm1Fwa N12 (RAG KO) mice (Taconic Biosciences) were housed under specific pathogen-free conditions in an Association for Assessment and Accreditation of Laboratory Animal Care [AALAC]-approved facility. At the end of each study, mice were euthanized by isoflurane overdose followed by cervical dislocation. All procedures conducted on animals were prospectively approved by the Abbvie Institutional Animal Care and Use Committee, and all mice were monitored by an attending veterinarian.

### Administration of minicircle vectors

Hydrodynamic injection of IL-23 expressing mc has been described [6, 7]. IL-23 and sham mc were purchased from System Biosciences, Inc. 3ug DNA was diluted in sterile Ringer’s solution equivalent to 10% mouse body weight and injected i.v. into B6 mice over ~5 seconds. IL-23 expression was confirmed in plasma at harvest by ELISA (R&D Systems or Meso Scale Discovery).

### Isolation of lamina propria cells

For consistency, the SI was measured and the most proximal third was harvested for flow cytometric analyses (regardless of differences in length). Fat and Peyer’s Patches were removed, and intestines were flushed with PBS and cut open longitudinally and into 1 cm segments. Mucus and epithelial cell removal were based on previously described methods [8]. Briefly, tissue segments were washed 20 min on a 200 rpm cell shaker in an HBSS solution of 5mM DTT (Sigma), followed by three 15 min washes in an HBSS solution of 5mM EDTA (Sigma). Segments were then digested with the mouse Lamina Propria Dissociation Kit used as directed (Miltenyi Biotec). After mechanical dissociation, samples were further mashed through a 70μM strainer.

### Flow cytometry

Cells were stained with the Abs indicated (BD Biosciences, Biolegend, and eBioscience). For cytokine staining, cells were incubated 3 hours with Protein Transport Inhibitor cocktail (eBioscience) prior to staining via the Transcription Factor Buffer Set (BD Biosciences). This kit was also used to assess Ki67 and transcription factors. The dump cocktail stain included Abs to B220, CD11b, CD11c, F4/80, Gr-1, and Ter119 (Biolegend). Dead cells were excluded from analysis using Fixable Live/Dead dye (Invitrogen), and CountBright beads (Life Technologies) were used to calculate cell numbers. Cytometry was performed on an LSRFortessa™ (BD Biosciences), and FlowJo (TreeStar) was used for data analysis.

### Small intestinal explant culture

1 cm SI sections were obtained (just distal to segment taken for LP cell isolation), and were cleaned and cut open as above. Sections were placed on ice in 1ml PBS containing 1x Antibiotic-Antimycotic (Gibco) during the harvest and were then transferred to 250ul IMDM GlutaMAX supplemented with 10% FBS, 1x Penicillin Streptomycin, and 2-Mercaptoethanol (Gibco) and incubated at 37°C for 24 hours. Supernatants were collected and cytokine production assessed by ELISA (R&D Systems or Meso Scale Discovery).

### Culture of isolated ILCs

LP cells were obtained and pooled from RAG KO mice, and ILC3 were enriched by negative selection with a cocktail of biotinylated Abs specific for CD11b, CD11c, F4/80, Gr-1, Ter119, NK1.1, FcεRI, EpCAM, and KLRG1 (Biolegend) followed by incubation with Anti-Biotin MicroBeads and magnetic separation as directed (Miltenyi Biotec). The negative fraction was stained with Fixable Live/Dead dye (Invitrogen), CD45 and CD90 specific Abs, and fluorescently conjugated streptavidin (to detect any remaining biotin labeled cells) (Biolegend). CD45+ CD90+ streptavidin- live cells (ILC3-enriched ILCs) were FACS sorted to ~97% purity using a FACSAria II (BD Biosciences). 20,000 cells per well were cultured 20 hours at 37°C in V-bottom plates (in IMDM media described above). Recombinant mouse cytokines concentrations used were 40ng/ml IL-23, 10ng/ml IL-2, and 40ng/ml TNFα (R&D Systems).

### TNFα neutralization

For *in vivo* TNFα neutralization, mice were injected i.p. with either PBS or 15mg/kg anti-mouse TNF IgG2c mAb (clone 8C11, AbbVie).

### Histology and immunohistochemistry

Duodenal segments were fixed in 10% buffered formalin and embedded in paraffin (FFPE) prior to sectioning and staining with H&E. 5 um FFPE sections were deparaffinized, rehydrated, and placed on a Leica Bond Rx immunohistochemical stainer for heat-activated antigen retrieval and sequential staining with Rabbit anti-human CD3 (Lab Vision), the Bond Polymer Refine Detection kit (Leica), and Deep Space Black Chromogen (Biocare Medical). Slides were then incubated with rabbit anti-IBA1 (Wako) and the Bond Polymer Refine Red Detection kit (Leica) and were counterstained with methyl-green. Isotype matched antibodies were used on serial tissue sections as negative controls.

### Statistical analysis

Graphed data were analyzed for statistical significance with Prism software (Graphpad). Unpaired two-tailed Student’s *t* tests were used to calculate p values, with <0.05 considered statistically significant (*p<0.05, **p<0.01, ***p<0.001, ****p<0.0001).

## Results and discussion

### Elevated systemic IL-23 induces GI inflammation and epithelial cell hyperplasia in healthy adult mice

**Fig 1:**
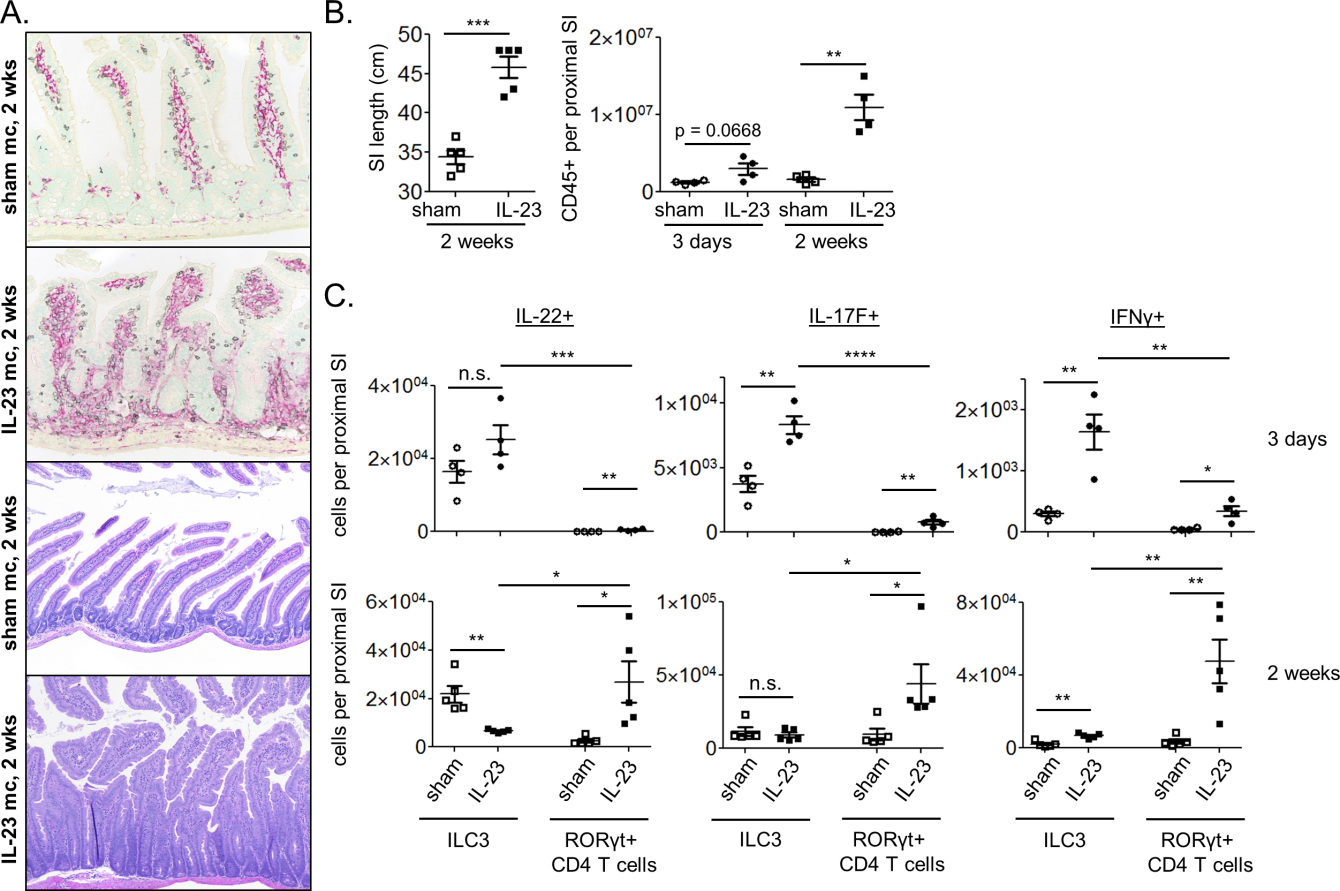
Chronic IL-23 elevation results in intestinal inflammation within the upper GI tract of wildtype B6 mice. Mice were injected with sham mc (open symbols) or IL-23 mc (filled symbols). (A) IHC (top) and H+E staining (bottom) of duodenal tissue. For IHC, CD3e+ staining (T cells) is black, and IBA-1 staining (macrophages) is red. (B) SI length 2 weeks post mc injection. (B-C) Flow cytometry was performed on LP cells from the proximal SI and the absolute numbers of (B) hematopoietic and (C) cytokine producing ILC3 and RORγt+ T cells were quantified (see S1B Fig for typical gating strategy). Scatter plots show means ± SEM for all mice from one of 2–4 similar experiments, 4–5 mice per group, with each symbol representative of a single mouse.

To induce high systemic IL-23, WT B6 mice were injected with either an IL-23 expressing mc (IL-23 mc) or an empty control vector (sham mc) and were assessed for intestinal pathology at 2, 4, and 8 weeks post-injection. SI inflammation and epithelial cell hyperplasia were observed histologically by 2 weeks post IL-23 mc injection, with the most dramatic pathology observed in the duodenum (Fig 1A). Overnight culture of SI explants revealed robust production of IL-22, IL-17F, and IFNγ (S1A Fig), and thickening and lengthening of the SI were grossly apparent (Fig 1B). Flow cytometric analysis of isolated LP cells revealed hematopoietic cell infiltration by 3 days post IL-23 mc injection, which became more dramatic by 2 weeks (Fig 1B). Although plasma IL-23 was sustained at levels similar to those previously described (S1B Fig) [7], duodenal adenoma formation was not observed at any timepoint assessed. It is possible that this reflects variance in microbial communities between facilities.

Though the levels of plasma IL-23 achieved in this model were quite high, IL-23 secretion was very low in SI tissue explant cultures (S1B Fig). This was not unexpected since the majority of plasmid uptake in hydrodynamic minicircle studies occurs in hepatocytes in the liver, with overexpressed soluble factors then entering the blood supply [9, 10]. It is unclear how the supraphysiologic plasma IL-23 levels (10ng/ml) achieved in this model compare to the localized tissue concentrations that would typically be produced by innate immune cells during a natural immune response. Therefore, the plasma levels of IL-23 produced in this model should not be compared directly with the levels observed in other IL-23 associated models of intestinal inflammation.

### ILC3 make up the majority of the early IL-23 induced intestinal cytokine response

ILC3 and Th17 cells can both produce IL-17F and IL-22 in response to IL-23, which in turn drives hematopoietic cell recruitment and proliferation of intestinal epithelial cells [11, 12]. Flow cytometric analysis of ILC3 and RORγt+ CD4 T cells (S1C Fig) in IL-23 mc treated mice revealed a hand off in cytokine production between the two cell types, with ILC3 making up the majority of cytokine producing cells at day 3, and RORγt+ CD4 T cells infiltrating and producing more cytokines by 2 weeks post IL-23 mc injection (Fig 1C; S1D Fig).

### Sustained IL-23 exposure induces IL-7R downregulation on activated ILC3 and a striking loss of these cells from the proximal GI tract

**Fig 2:**
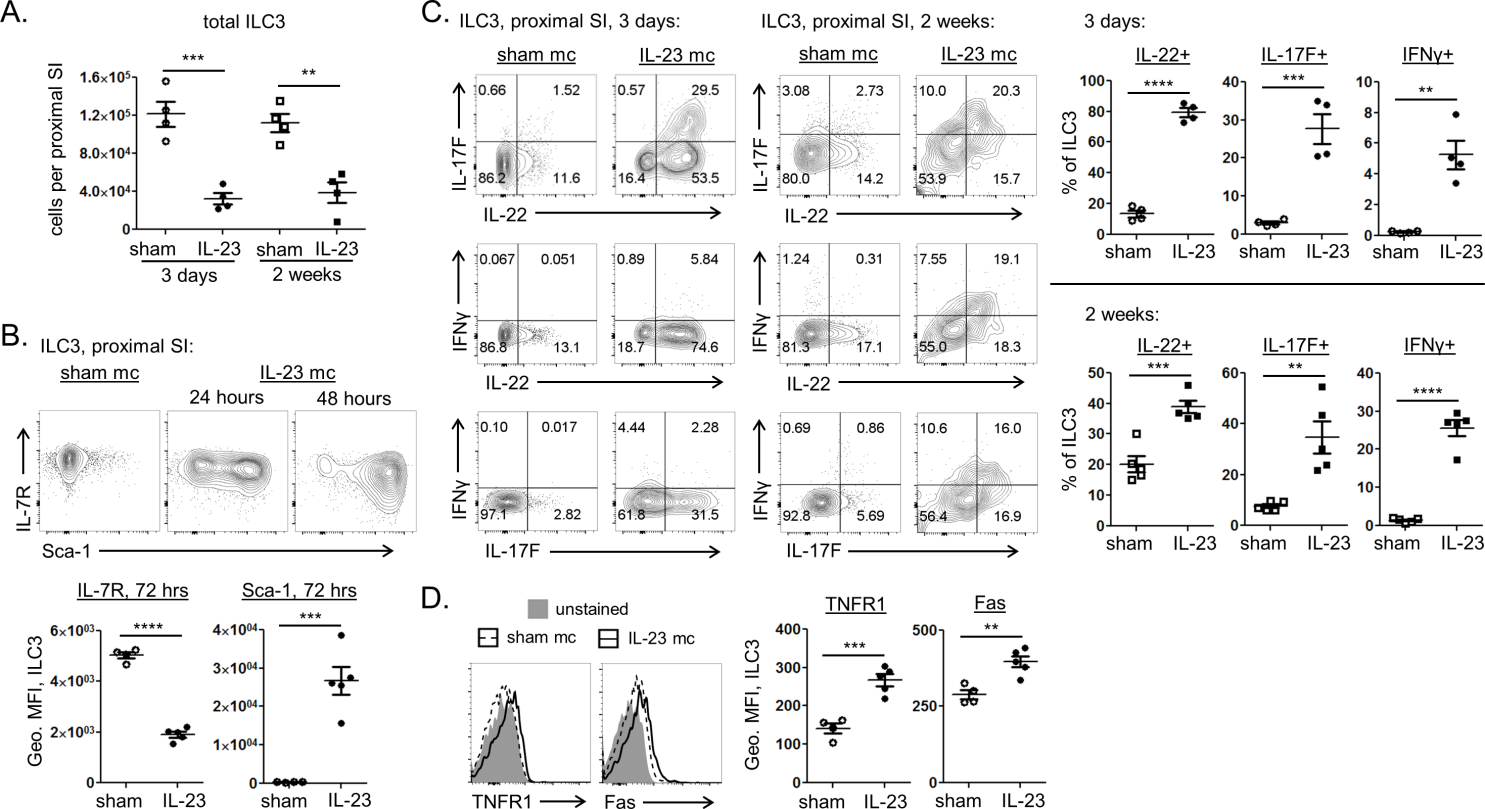
ILC3 are rapidly activated and depleted from the proximal small intestine of IL-23 mc injected mice. Mice were injected with sham mc (open symbols) or IL-23 mc (filled symbols), and flow cytometry was performed on LP cells from the proximal SI. (A) The absolute number of ILC3 (defined as CD45+ CD90+ CD11b- CD11c- F4/80- Gr-1- Ter119- B220- CD3e- RORγt+ cells; see S1B Fig for typical gating) is shown. (B) Top: representative staining shows protein expression at 24 and 48 hours post mc injection. Bottom: compiled staining intensities are depicted as geometric MFI at 72 hours post mc injection. (C) Representative staining shows cytokine expression three days and 2 weeks post mc injection (left) along with compiled data (right). (D) Expression of cell death associated receptors by ILC3 is shown three days post mc injection. Representative and compiled staining intensities are shown. Scatter plots show means ± SEM for all mice from one of 2–4 similar experiments, 4–5 mice per group, with each symbol representative of a single mouse.

**Fig 3:**
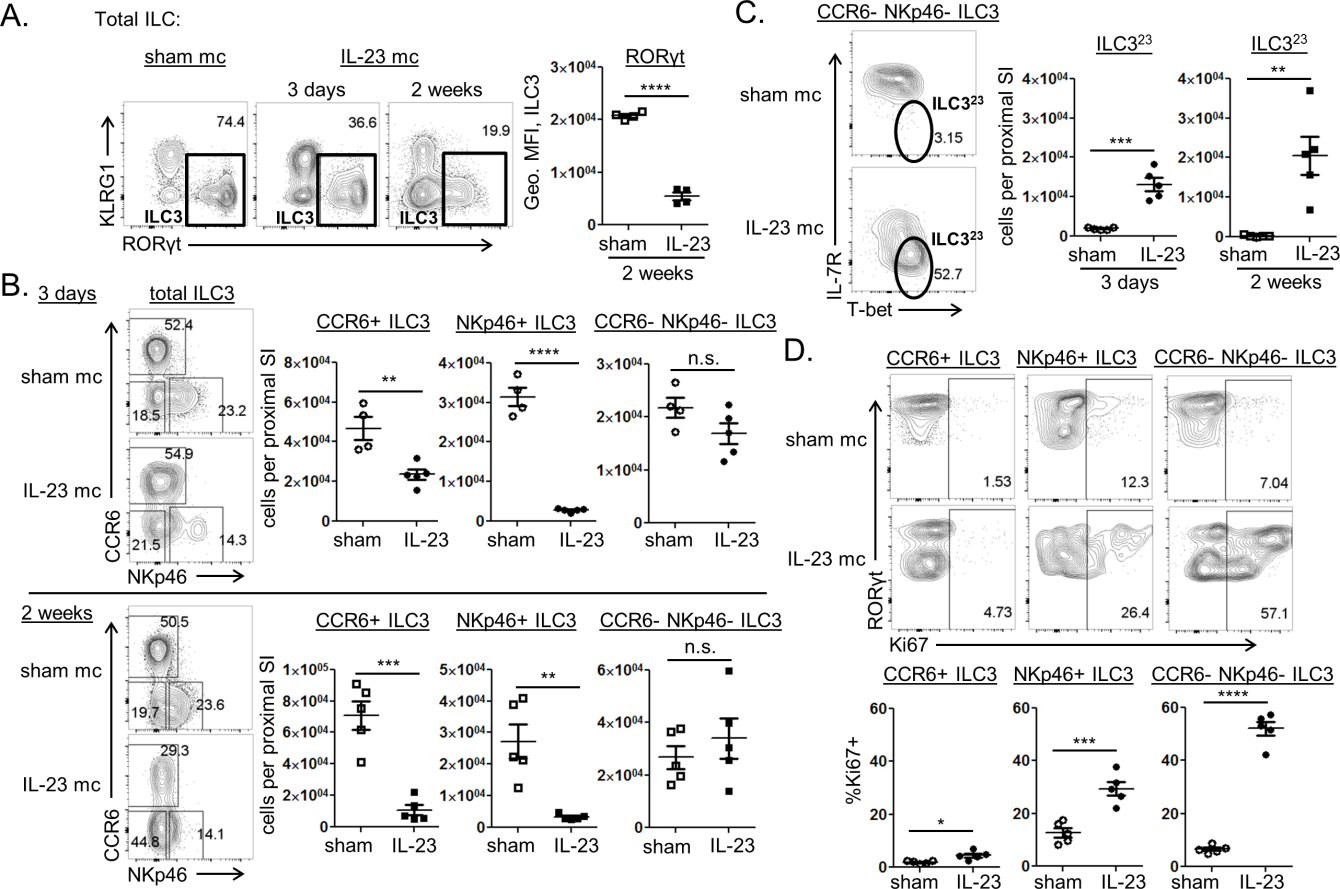
Both CCR6+ and NCR+ ILC3 subsets are reduced following IL-23 stimulation, and remaining ILC3 are phenotypically distinct. Mice were injected with sham mc (open symbols) or IL-23 mc (filled symbols), and flow cytometry was performed on LP cells from the proximal SI. (A) Representative staining shows the frequency of ILC3 amongst total ILC at 3 days and 2 weeks post mc injection, with compiled geometric MFI of RORγt expression by ILC3 shown. (B) Representative staining shows the frequency of ILC3 subsets amongst total ILC3 at 3 days (top) and 2 weeks (bottom) post mc injection with compiled absolute numbers. (C) Representative staining shows the frequency of IL-23 mc elicited ILC3 (ILC3^23^; IL-7R- T-bet+) amongst CCR6- NCR- ILC3 at 3 days post mc injection, with compiled absolute numbers of these cells shown at 3 days and 2 weeks post mc injection. (D) Representative staining shows the frequency of Ki67+ cells amongst ILC3 subsets, with compiled data at 3 days post mc injection. Scatter plots show means ± SEM for all mice from one of at least 3 similar experiments, 4–5 mice per group, with each symbol representative of a single mouse.

Though the number of cytokine producing ILC3 increased soon after IL-23 mc injection (Fig 1C), enumeration of total ILC3 in the upper SI revealed a rapid and lasting reduction of ILC3 after 3 days of IL-23 exposure (Fig 2A). This profound loss of LP ILC3 could not be explained by trafficking of these cells out of the intestine, since only a subtle increase in ILC3 was observed in mesenteric and pancreatic lymph nodes (S2A Fig). The recently described ability of ILC3 to convert into other ILC subsets [13–15] also failed to account for the rapid loss of ILC3 in this model, since no concurrent increase in ILC1 or ILC2 populations was observed within the upper GI of IL-23 mc treated mice at 3 days post IL-23 mc injection (S2B Fig). Flow cytometric examination of the early ILC3 response to IL-23 revealed rapid upregulation of Sca-1, a marker previously shown to be expressed by some activated ILC3populations (Fig 2B) [16, 17]. Interestingly, we also observed a rapid and profound drop in surface IL-7R expression on activated ILC3 (Fig 2B), simultaneous to robust production of IL-22 and IL-17F (Fig 2C). Since IL-7 is fundamental for the homeostasis and maintenance of ILC populations [18], this may in part explain the decrease in ILC3 population size. Further examination of the remaining intestinal ILC3 revealed increased surface expression of the death-domain containing receptors Fas and TNFR1 (Fig 2D), suggesting a heightened susceptibility of these cells to apoptosis. IL-23 stimulated ILC3 also exhibited a progressive reduction in RORγt expression magnitude, further implying decreased fitness of the subset (Fig 3A). This decline in RORγt expression may in fact be due to the drop in IL-7R expression, since previous studies have found IL-7 signals to stabilize RORγt expression by ILC3 [13]. A similar reduction of ILC3 has been described during IBD and chronic viral infection in mice, primates, and humans [15, 19–22], suggesting IL-23 or other chronic inflammatory stimuli may drive activation induced death of these cells.

### Both CCR6+ and NCR+ ILC3 subsets are dramatically reduced by prolonged IL-23 exposure, and a phenotypically distinct ILC3 population emerges

Multiple ILC3 subsets have been described within the intestine, with each playing distinct but overlapping roles during an immune response. While all ILC3 express RORγt and produce IL-22 upon activation, cells within the CCR6+ ILC3 population can additionally produce IL-17F, express MHC-II, and participate in regulatory interactions with T cells, while CCR6- ILC3 can express NKp46 and T-bet and produce IFNγ during inflammation [4]. Since cytokine production by chronically IL-23-activated ILC3 shifted from primarily IL-22 and IL-17F secretion to an increasingly IFNγ associated response over time (Fig 2C; Fig 1C), we hypothesized that specific ILC3 subsets were being depleted during the response to IL-23 mc. Examination of ILC3 populations within IL-23 mc treated mice revealed both CCR6+ and NKp46+ ILC3 to be lost upon IL-23 stimulation (Fig 3B). Interestingly, the CCR6- NKp46- ILC3 population was maintained throughout IL-23 mc treatment, and a subset of these cells exhibited a dramatic increase in T-bet expression concurrent to their loss of IL-7R (Fig 3B and 3C). These CCR6- NKp46- IL-7R- T-bet+ ILC3 maintained low level expression of RORγt, which in addition to their lack of IL-7R expression, distinguished them from previously described Nkp46+ T-bet+ IL-7R+ inflammatory ILC3 and IL-7R+ RORγt- “ex-RORγt+” ILC populations (S2C Fig) [14, 15]. They were also phenotypically distinct from intestinal NK cells and other KLRG1-RORγt- ILC (“other ILC1”) that become increased in this model after two weeks of continual IL-23 stimulus (S2C and S2B Fig). We here refer to these cells as IL-23 mc elicited ILC3 (ILC3^23^ for ease of notation) since they appear during the sustained IL-23 stimulus intrinsic to this model. Examination of Ki67 staining in ILC3 subsets revealed division to be most robust within the CCR6- NKp46- ILC3 subset (Fig 3D). This suggests that the increase in ILC3^23^ may result from IL-23 driven expansion of a pre-existing NKp46- CCR6- population, perhaps in an attempt to replenish the lost population of NKp46+ ILC3. Indeed, intestinal IL-23 has previously been associated with T-bet expression in CCR6- ILC3, and T-bet+ CCR6- ILC3 are thought to be the direct precursor to NKp46+ ILC3 [14]. Alternatively, NKp46+ ILC3 may convert into ILC3^23^ upon IL-23 stimulus, as fate-mapping studies have shown that NKp46+ ILC3 can lose NKp46 expression under certain circumstances [23].

It should be noted that, though ILC3 are also known to produce IL-17A in response to various infections and inflammatory stimuli, we did not observe appreciable IL-17A production in this model at any timepoint assessed (S1E Fig). Additional cytokines, costimulatory signals, or pathogen-associated factors may be required in this model to induce IL-17A production in these cells.

### TNFα neutralization inhibits IL-23 driven ILC3 function in vivo, but it does not affect the loss of these cells from the proximal GI tract

**Fig 4:**
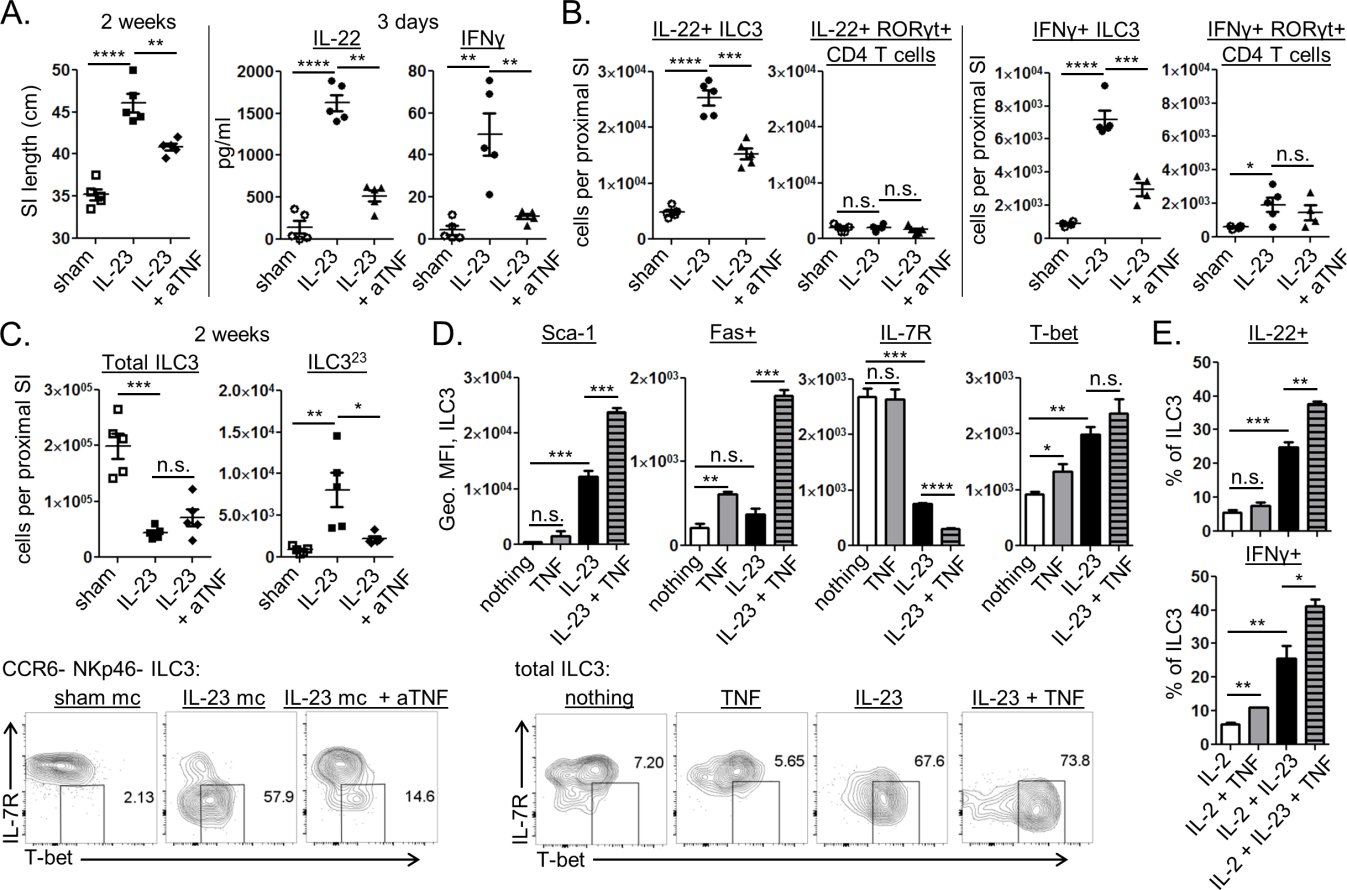
TNFα promotes cytokine production by ILC3 and ILC3^23^ formation *in vivo*, and synergizes with IL-23 to directly activate ILC3 *in vitro*. (A-C) Mice were injected with sham mc (open symbols) or IL-23 mc (filled symbols), with some groups receiving anti-TNFα (or PBS) as indicated. (A) SI length 2 weeks post mc injection (left), and cytokine secretion from SI explants harvested 3 days post mc injection (right) are shown. (B) Flow cytometry was performed on LP cells from the proximal SI three days post mc injection. Absolute numbers of cytokine producing lymphocytes are shown. (C) Flow cytometry was performed on LP cells from the proximal SI 2 weeks post mc injection. Top: absolute numbers are shown. Bottom: representative staining shows the frequency of ILC3^23^ amongst CCR6- NCR- ILC3. (D-E) FACS purified ILCs enriched for ILC3 (~90% RORγt+) were sorted from the proximal SI LP of RAG KO mice and cultured overnight with the indicated cytokines prior to flow cytometry. (D) Protein expression by ILC3 is shown for triplicate wells with staining intensity depicted as geometric MFI (top), and representative IL-7R and T-bet staining is shown for total ILC3 (bottom). (E) Cytokine expression by ILC3 is shown for triplicate wells treated with protein inhibitors for the last 3 hours of culture. Scatter plots show means ± SEM for all mice from one of 2–3 similar experiments, 4–5 mice per group, with each symbol representative of a single mouse. Bar graphs show means ± SEM for triplicate wells from one of two similar experiments.

TNFα can be made by a variety of activated hematopoietic and non-hematopoietic cells within the intestine, and TNFα neutralization has proven to be an effective therapeutic approach for the treatment of IBD [24]. Since IL-23 mc treatment resulted in intestinal inflammation and deregulated intestinal epithelial cell growth, we wondered if TNFα blockade might affect any of this pathology. To test this hypothesis, mice were treated with anti-TNFα starting one day prior to IL-23 mc injection and continuing twice weekly until the time of harvest. By 2 weeks we observed a reduction in SI lengthening in anti-TNFα treated mice, suggesting an overall benefit of TNFα blockade in restoring intestinal homeostasis (Fig 4A). Surprisingly, SI explant cultures revealed a dramatic reduction in intestinal IFNγ and IL-22 levels as early as 3 days post IL-23 mc injection (Fig 4A). This suggested TNFα neutralization to be inhibiting aspects of the early IL-23 driven ILC3 response. Enumeration of cytokine producing ILC3 revealed that TNFα blockade diminished the numbers of IL-22+ and IFNγ+ ILC3 at a timepoint prior to substantial production of these cytokines by RORγt+ CD4 T cells (Fig 4B). While TNFα blockade decreased cytokine production by ILC3, it was unable to reverse the overall loss of ILC3 observed in this model (Fig 4C). Interestingly, the ILC3^23^ population was reduced in anti-TNFα treated mice, suggesting TNFα may be a driver of this cell type (Fig 4C).

Though we did not endeavor to pinpoint the source of the TNFα contributing to ILC3 activation in this model, it should be noted that in addition to several other cell types, activated ILC3 can themselves produce TNFα during intestinal inflammation [5]. We indeed observed TNFα production by ILC3 at both early and late timepoints in this model (S1F Fig), making it possible that autocrine TNFα production plays a role in our observations. Further studies would be required to explicitly resolve the source of the TNFα in this model.

### TNFα and IL-23 synergize to directly promote ILC3 activation and cytokine production in vitro

The ability of TNFα neutralization to inhibit ILC3 cytokine production and the appearance of ILC3^23^ cells *in vivo* made us wonder if TNFα was acting directly on ILC3 to promote IL-23 driven activation of these cells. To test this hypothesis, we FACS purified an ILC3-enriched population of CD90.2+ ILCs (~90% RORγt+ post FACS sort) from the upper SI LP of RAG KO mice and stimulated them overnight with IL-23, TNFα, or a combination of both. While TNFα stimulus alone had little to no effect on these cells, IL-23 and TNFα synergistically promoted downregulation of IL-7R, upregulation of Sca-1 and Fas, and production of IL-22 and IFNγ (Fig 4D and 4E). IL-23 driven T-bet upregulation was also observed by cultured ILC3, but adding TNFα did not significantly enhance this increase *in vitro* (Fig 4D). These data are the first to show that activated ILC3 are directly responsive to TNFα and that TNFα can synergize with IL-23 to promote ILC3 function both *in vitro* and *in vivo*.

### Closing remarks

In this study we explored the intestinal ILC3 response to sustained IL-23 stimulus in the absence of any overt infection. We found that ILC3 populations within the proximal small intestine are rapidly activated in response to IL-23, but that the size of the ILC3 population is dramatically reduced upon such stimulus. While a decrease in ILC3 has been observed in certain disease states [15, 19–22], this is the first description of a loss of ILC3 after sustained IL-23 exposure. Future studies would be needed to determine whether ILC3 numbers would normalize if IL-23 treatment were ceased and how long such a recovery might take.

While conventional ILC3 populations were rapidly depleted from the upper GI tract upon IL-23 mc treatment, an IL-7R- T-bet+ ILC3 population emerged that we referred to as IL-23 mc elicited ILC3 (denoted ILC3^23^). TNFα neutralization, which is widely used as a therapeutic strategy for IBD, diminished the ILC3 cytokine response and the emergence of ILC3^23^ cells after IL-23 stimulus; however it did not reverse the overall loss of classical ILC3 populations that occurred in our model. This suggests that TNFα promotes IL-23 driven ILC3 activation, but does not drive the loss of conventional ILC3 that occurs in mice injected with IL-23 mc. *In vitro* ILC cultures further illustrated synergy between IL-23 and TNFα in driving activation and cytokine production by ILC3. It is unclear whether anti-TNF mediated blockade of ILC3 activation would be harmful or beneficial to an inflamed host, since moderate IL-22 levels are thought to benefit the GI tract, while high levels of IL-22 and IFNγ have been associated with tumor formation and intestinal damage [4, 7]. Indeed previously described IFNγ producing ILC3 populations are known to be pathogenic during Salmonella infection and experimental colitis [13, 14]. Further studies would be needed to determine whether or not ILC3^23^ cells are similarly pathogenic and if they arise in other models of IL-23 associated intestinal inflammation.

Interestingly, the effects of IL-23 minicircle injection were most apparent in the proximal GI tract, as had been reported in a previous study [7]. Although the reason for this spatial distinction remains unclear, studies in IL-23R reporter mice reveal IL-23R expression to be highest in the small intestine, with ILC3 representing the vast majority of IL-23R+ populations within this tissue at steady state [3]. It then makes sense that the earliest responders to IL-23 in an otherwise healthy host would be ILC3 populations within the small intestine. Teleologically speaking, it is possible that IL-23 exposure in the proximal intestine would be considered significantly out of the ordinary to the immune system, perhaps representing exposure to a food-borne parasite and a threat that must be swiftly eliminated. Conversely, the colon is home to a host of microbial communities and associated TLR ligands, so regulatory mechanisms at play in the colon that serve to prevent an excessive immune response to commensals may also prevent immune cells in this region from responding strongly to IL-23 alone. Further studies will be required to determine how commensal bacteria affect the intestinal immune response in this model. It may also be interesting to overexpress other cytokines associated with ILC3 activation, such as IL-1β, to determine whether the ILC3 population shrinkage and phenotypic changes observed here are specific to prolonged IL-23 stimulus or represent a broader paradigm of the ILC3 response to chronic activation.

ILC3 are constitutively responsive to IL-23 and are present at high numbers in healthy intestine [3–5]. Because of this, these cells are likely key players in the switch between intestinal homeostasis and IL-23 associated IBD. It is intriguing to speculate that the loss of ILC3 observed during IBD [15, 22] might be driven by an overactive IL-23 response in a genetically susceptible individual. Studies in mice suggest that a lack of ILC3 can be detrimental to a host, as IL-22 produced by ILC3 is important for maintenance of the intestinal epithelium, and ILC3 depletion has been associated with low-grade systemic inflammation [25, 26]. Perhaps ILC3 loss due to chronic IL-23 stimulation precedes more overt inflammation and may represent an important step in the progression to IBD.

## Supporting information

S1 Fig Cytokine production in IL-23 mc injected mice and gating strategy for simultaneous analysis of ILC3 and RORγt+ T cell populations within the SI LP. Mice were injected with sham mc (open symbols) or IL-23 mc (filled symbols), and flow cytometry was performed on LP cells from the proximal SI. (A) Cytokine secretion from 24 hour SI explants. (B) IL-23 secretion measured in blood plasma (left) and 24 hour SI explants (right). (C) Gating strategy for simultaneous analysis of ILC3 and RORγt+ CD4+ T cells within the proximal SI LP. Representative staining from a sham mc injected mouse, previously gated on live single cells, is shown. (D) The absolute number of RORγt+ CD4+ T cells is shown for several mice at 3 days and 2 weeks post mc injection. (E) IL-17A secretion from 24 hour SI explants (left) and representative staining for IL-17A and IL-22 expression by ILC3 3 days and 2 weeks post mc injection (right). (F) Compiled TNFα expression data is shown for ILC3 from the proximal SI 3 days and 2 weeks post mc injection. Scatter plots show means ± SEM for all mice from one of at least three similar experiments, 4–5 mice per group, with each symbol representative of a single mouse.
(PDF)

S2 Fig Loss of ILC3 from the proximal SI LP cannot be explained by trafficking to lymph nodes or conversion to other ILC subsets, and ILC323 are phenotypically distinct from intestinal NK cells. (A) Mice were injected with sham mc (open symbols) or IL-23 mc (filled symbols), and flow cytometry was performed on the indicated lymph nodes (LNs), which had been dissociated through fine mesh prior to staining for flow cytometry. The absolute number of ILC3 is shown as compiled data from multiple mice at 3 days post mc injection. (B-C) Mice were injected with sham mc (open symbols) or IL-23 mc (filled symbols), and flow cytometry was performed on LP cells from the proximal SI. (B) Representative gating (top) and the absolute numbers of ILC1,ILC2, and ILC3 subsets (bottom) are shown compiled from several mice at 3 days and 2 weeks post mc injection (see S1C Fig for additional gating strategy). (C) Left: representative staining shows the frequency of NKp46+ NK1.1+ cells amongst ILC3^23^ (IL-7R- T-bet+ CCR6- NCR- ILC3, top) and KLRG1- RORγt- ILC1 subsets (bottom) at 2 weeks post IL-23 mc injection. Right: protein expression by NK cells (NK1.1+ NKp46+ ILC1) and ILC3^23^ is shown compiled from several mice at 2 weeks post IL-23 mc injection, with staining intensity depicted as geometric MFI. Scatter plots show means ± SEM for all mice from one of 2–3 similar experiments, 4–5 mice per group, with each symbol representative of a single mouse.
(PDF)
