# Supplementary figures and images for: Continuous IL-23 stimulation drives ILC3 depletion in the upper GI tract and, in combination with TNFα, induces robust activation and a phenotypic switch of ILC3
Source: PLoS One. 2017 Aug 8;12(8):e0182841. doi: 10.1371/journal.pone.0182841 (PMC5549730; doi:10.1371/journal.pone.0182841)

Figure S1

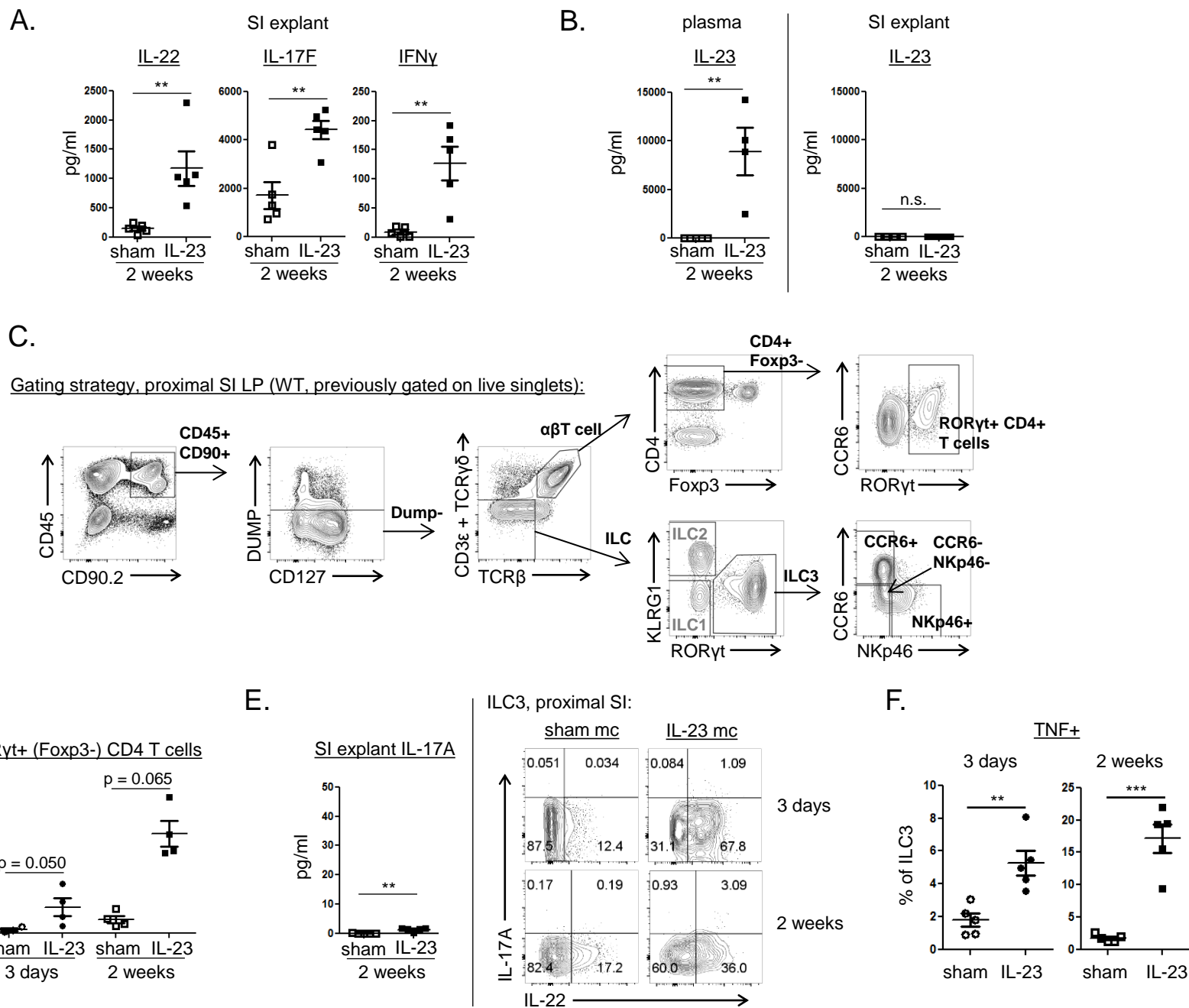

Supplement: S1 Fig — Mice were injected with sham mc (open symbols) or IL-23 mc (filled symbols), and flow cytometry was performed on LP cells from the proximal SI. (A) Cytokine secretion from 24 hour SI explants. (B) IL-23 secretion measured in blood plasma (left) and 24 hour SI explants (right). (C) Gating strategy for simultaneous analysis of ILC3 and RORγt+ CD4+ T cells within the proximal SI LP. Representative staining from a sham mc injected mouse, previously gated on live single cells, is shown. (D) The absolute number of RORγt+ CD4+ T cells is shown for several mice at 3 days and 2 weeks post mc injection. (E) IL-17A secretion from 24 hour SI explants (left) and representative staining for IL-17A and IL-22 expression by ILC3 3 days and 2 weeks post mc injection (right). (F) Compiled TNFα expression data is shown for ILC3 from the proximal SI 3 days and 2 weeks post mc injection. Scatter plots show means ± SEM for all mice from one of at least three similar experiments, 4–5 mice per group, with each symbol representative of a single mouse. (PDF) [file pone.0182841.s001.pdf]
