# Supplementary material for: Continuous IL-23 stimulation drives ILC3 depletion in the upper GI tract and, in combination with TNFα, induces robust activation and a phenotypic switch of ILC3
Source: PLoS One. 2017 Aug 8;12(8):e0182841. doi: 10.1371/journal.pone.0182841 (PMC5549730; doi:10.1371/journal.pone.0182841)
Supplement: S2 Fig — (A) Mice were injected with sham mc (open symbols) or IL-23 mc (filled symbols), and flow cytometry was performed on the indicated lymph nodes (LNs), which had been dissociated through fine mesh prior to staining for flow cytometry. The absolute number of ILC3 is shown as compiled data from multiple mice at 3 days post mc injection. (B-C) Mice were injected with sham mc (open symbols) or IL-23 mc (filled symbols), and flow cytometry was performed on LP cells from the proximal SI. (B) Representative gating (top) and the absolute numbers of ILC1,ILC2, and ILC3 subsets (bottom) are shown compiled from several mice at 3 days and 2 weeks post mc injection (see S1C Fig for additional gating strategy). (C) Left: representative staining shows the frequency of NKp46+ NK1.1+ cells amongst ILC323 (IL-7R- T-bet+ CCR6- NCR- ILC3, top) and KLRG1- RORγt- ILC1 subsets (bottom) at 2 weeks post IL-23 mc injection. Right: protein expression by NK cells (NK1.1+ NKp46+ ILC1) and ILC323 is shown compiled from several mice at 2 weeks post IL-23 mc injection, with staining intensity depicted as geometric MFI. Scatter plots show means ± SEM for all mice from one of 2–3 similar experiments, 4–5 mice per group, with each symbol representative of a single mouse. (PDF) [file pone.0182841.s002.pdf]

Figure S2

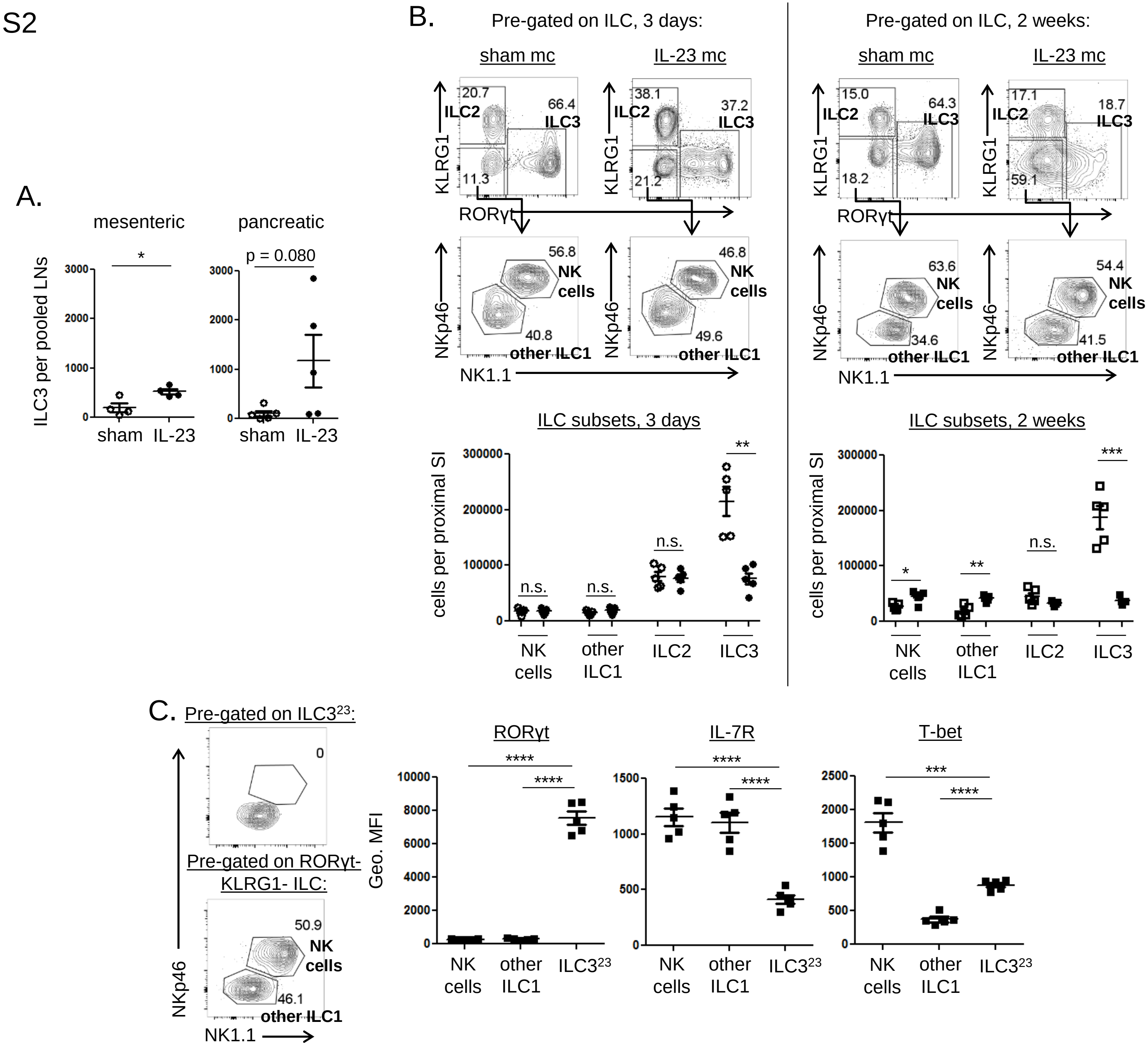

A.
mesenteric
pancreatic
ILC3 per pooled LNs
*
p = 0.080
3000
2000
1000
0
sham
IL-23
B.
Pre-gated on ILC, 3 days:
Pre-gated on ILC, 2 weeks:
sham mc
IL-23 mc
KLRG1
RORγt
ILC2
ILC3
20.7
66.4
11.3
38.1
37.2
21.2
15.0
64.3
18.2
17.1
18.7
59.1
NKp46
NK1.1
NK cells
other ILC1
56.8
40.8
46.8
49.6
63.6
34.6
54.4
41.5
ILC subsets, 3 days
ILC subsets, 2 weeks
cells per proximal SI
300000
200000
100000
0
n.s.
**
***
*
NK cells
other ILC1
ILC2
ILC3
C.
Pre-gated on ILC3²³:
0
Pre-gated on RORγt- KLRG1- ILC:
50.9
46.1
RORγt
IL-7R
T-bet
Geo. MFI
****
***
10000
8000
6000
4000
2000
1500
1000
500
2500
ILC3²³
